# The complete genome of klassevirus – a novel picornavirus in pediatric stool

**DOI:** 10.1186/1743-422X-6-82

**Published:** 2009-06-18

**Authors:** Alexander L Greninger, Charles Runckel, Charles Y Chiu, Thomas Haggerty, Julie Parsonnet, Donald Ganem, Joseph L DeRisi

**Affiliations:** 1 Howard Hughes Medical Institute, Departments of Medicine, Biochemistry, and Microbiology, University of California, San Francisco, California 94143, USA; 2 Departments of Laboratory Medicine and Medicine, Division of Infectious Diseases, University of California, San Francisco, California 94143, USA; 3 Department of Medicine, Division of Infectious Diseases, Stanford University, Stanford, California 94305, USA

## Abstract

**Background:**

Diarrhea kills 2 million children worldwide each year, yet an etiological agent is not found in approximately 30–50% of cases. Picornaviral genera such as enterovirus, kobuvirus, cosavirus, parechovirus, hepatovirus, teschovirus, and cardiovirus have all been found in human and animal diarrhea. Modern technologies, especially deep sequencing, allow rapid, high-throughput screening of clinical samples such as stool for new infectious agents associated with human disease.

**Results:**

A pool of 141 pediatric gastroenteritis samples that were previously found to be negative for known diarrheal viruses was subjected to pyrosequencing. From a total of 937,935 sequence reads, a collection of 849 reads distantly related to Aichi virus were assembled and found to comprise 75% of a novel picornavirus genome. The complete genome was subsequently cloned and found to share 52.3% nucleotide pairwise identity and 38.9% amino acid identity to Aichi virus. The low level of sequence identity suggests a novel picornavirus genus which we have designated klassevirus. Blinded screening of 751 stool specimens from both symptomatic and asymptomatic individuals revealed a second positive case of klassevirus infection, which was subsequently found to be from the index case's 11-month old twin.

**Conclusion:**

We report the discovery of human klassevirus 1, a member of a novel picornavirus genus, in stool from two infants from Northern California. Further characterization and epidemiological studies will be required to establish whether klasseviruses are significant causes of human infection.

## Background

Picornaviruses are positive-sense ssRNA viruses consisting of eight classical genera and six new proposed genera. They share a common genomic organization with a long 5' untranslated region (UTR) (500–800 nt) containing an internal ribosome entry site (IRES), a single ORF encoding a polyprotein that is proteolytically processed, and a short 3' UTR followed by a polyA tail [1]. Major differences among picornaviruses, among others, include the secondary structure of the 5' UTR and IRES and a VP0 capsid protein that is either cleaved into VP4 and VP2 or remains intact.

Kobuvirus is a genus in the family Picornavirus. There are three known kobuviruses: Aichi virus, bovine kobuvirus, and porcine kobuvirus [2-4]. All three have been discovered in stool specimens, with Aichi virus associated with non-bacterial human gastroenteritis, typically associated with oyster consumption [5]. Though originally isolated in Japan, Aichi virus has been found over a broad geographical range covering Asia, the Americas, and Europe [5,6]. All kobuviruses share the typical picornavirus genomic organization with genome sizes ranging from 8210–8374 nt. In addition to having a uncleaved VP0 capsid protein, kobuviruses have 3 highly conserved stem-loop structures in the first 120 nt of their 5' UTR which have been shown to be required for viral replication and encapsidation in Aichi virus [7,8].

Recently, pyrosequencing of stool samples from patients with acute flaccid paralysis from Pakistan was recently used to identify cosavirus, a new proposed picornaviral genus [9]. In this study, we report the discovery of a novel human picornavirus genus in two twins through pyrosequencing. We also report whole genome recovery and initial PCR screening for the novel picornavirus.

## Results

### Pyrosequencing of genome of novel picornavirus genus

**Figure 1:**
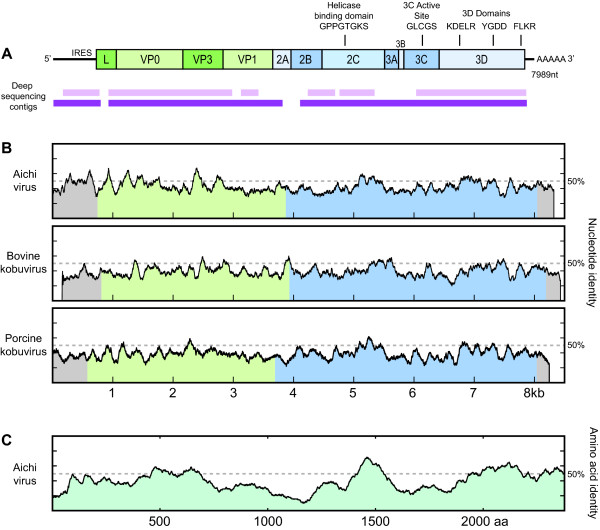
**A. Genome organization of human klassevirus 1**. Conserved picornaviral domains present in klassevirus are noted. Pyrosequencing contigs that align to Aichi virus by TBLASTX with an E-value of less than 10^-6 ^covered more than 75% of the genome (light purple). Pyrosequncing contigs that align to the human klassevirus 1 genome by BLASTN with an E-value of less than 10^-6 ^covered more than 95% of the full genome. B. Scanning nucleotide pairwise identity using a 100-bp window is depicted for Aichi virus, bovine kobuvirus, and porcine kobuvirus. C. Scanning amino acid pairwise identity using a 100-bp window versus Aichi virus.

As part of an ongoing investigation of pediatric gastroenteritis from Northern California, we identified 141 stool samples that were negative for viral detection by specific PCR for 7 stool viruses (adenovirus, astrovirus, calicivirus, rotavirus, enterovirus, cardiovirus, parechovirus) and Virochip, a pan-viral microarray. 141 samples were negative by array and PCR and were subjected to two sequencing runs on a Genome Sequencer FLX without molecular bar-coding. The two sequencing runs gave 937,935 filter pass reads with an average length of 241.7 bp, ranging from 32–503 bp. Of these, 849 reads had an E-value of less than 1e-6 against the Aichi virus genome by TBLASTX. Reads that aligned to Aichi virus assembled into approximately 75% of an expected ~8 kb genome (Figure 1). To identify the origin of the Aichi virus-like reads, reads were used to design primers [454A1F/454A2R, see Additional file 1] to screen amplified cDNA libraries of the original 141 samples. One sample (02394-01) was found to be positive with a 342 bp amplicon that matched the sequence recovered by pyrosequencing.

Given gaps in sequencing coverage and small picornavirus genome size, the pyrosequencing reads were used to design primers for subsequent amplification of the genome from sample 02394-01 total RNA [see Additional file 1]. RT-PCR was used to generate overlapping amplicons, which were cloned and subjected to Sanger sequencing. The 3' end of the genome was recovered by 3' RACE, while the 5' end of the genome was recovered by multiple iterations of 5' RACE using MLV and TTH reverse transcriptase from the most 5' pyrosequencing read that aligned to Aichi virus, approximately 250 nucleotides from the 5' end of the genome.

### Genome of novel picornavirus

The complete genome of the novel picornavirus is 7989 nt, excluding the poly-A tail [GenBank GQ184145]. A large ORF of 7113 nucleotides, encoding a 2371 amino acid potential polyprotein precursor, is flanked by a 5' UTR of 718 nt and a 3'UTR of 158 nt and poly(A) tail. The base composition of the coding region is 17.8% A, 36.0% C, 20.7% G, and 25.5% U. The genome shares 52.3%, 49.9%, and 49.8% pairwise nucleotide identity with Aichi virus, bovine kobuvirus, and porcine kobuvirus (Figure 1). The P1, P2, and P3 coding region has 38.0%, 34.8%, and 43.3% pairwise amino acid identity versus Aichi virus, suggesting it qualifies as a new picornavirus genus. We are provisionally naming this viral genus Klassevirus for *k*obu-*l*ike viruses *a*ssociated with *s*tool and *se*wage.

### 5' UTR

The 5' UTR of human klassevirus 1 is approximately the same length as that of Aichi virus (718 vs 745 nucleotides, respectively). The latter two-thirds of the UTR comprising the IRES aligns with 68% identity to Aichi virus, the highest identity of any area of the genome. However, the first 250 nucleotides of human klassevirus 1 have only 52% pairwise identity to Aichi virus and do not align to any sequence in GenBank. The first 120 nucleotides of Aichi virus comprising stem-loops A, B, and C (SL-A/B/C) have been shown to be critical for viral replication and encapsidation and the first 50 nucleotides of all previously identified kobuvirus genomes are very highly conserved.

**Figure 2:**
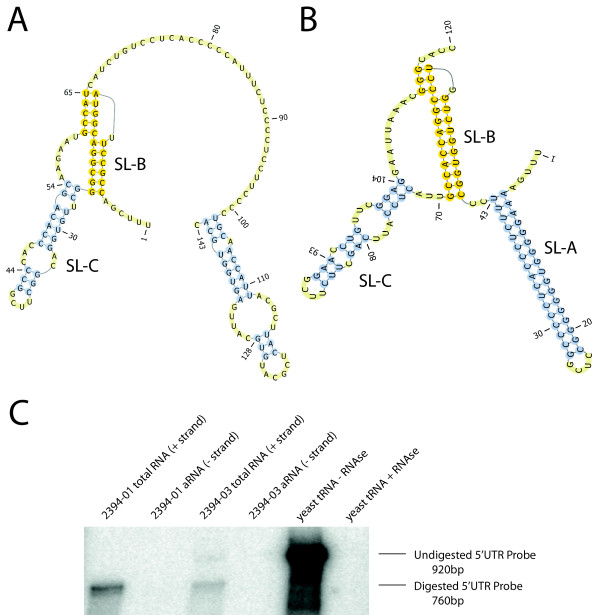
**A. Predicted RNA secondary structure of first 143 bp of 5' UTR of klassevirus using pknotsRG from Bielefeld University**. B. Predicted RNA secondary structure of first 120 bp of 5' UTR of Aichi virus using pknotsRG. The first 100 bp of Aichi virus, bovine kobuvirus, and porcine kobuvirus 5' UTRs are very conserved and have been shown to be critical for viral replication and encapsidation. C. RNAse protection experiment to show divergent klassevirus 5' UTR is contiguous. A 920-bp radiolabeled probe consisting of 760 bp of human kobuvirus 2 5' UTR flanked on each side by 80 bp of bacterial vector sequence was hybridized to stool total RNA, (-)-stranded kobuvirus, or nonsensical yeast tRNA, and digested by RNAse A/T1.

To rule out aberrant or chimeric amplification products during the recovery of the 5' UTR, RNAse protection was used to demonstrate that the initial 250 nt of the 5' UTR is on the same strand as the subsequent 500 nt that aligns to Aichi virus (Figure 2). Secondary structure analysis of the first 140 nt of human klassevirus 1 demonstrated some structural homology to Aichi virus 5' UTR secondary structure. Specifically, several stem-loop and pseudoknot structures are apparent in the first 100 bp, and no enterovirus/rhinovirus-like cloverleaf structures are recognized. However, no SL-A structure was found and the sequence context of the SL-B and SL-C structures are divergent with respect to Aichi virus (Figure 2).

**Figure 3:**
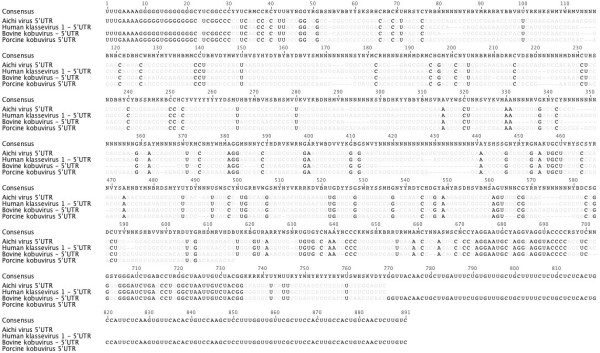
**Alignment of klassevirus and kobuvirus 5' UTRs**. The latter 500 bp of klasssevirus 5' UTR aligns with 69% identity to Aichi virus. We were unable to recover the conserved SL-A sequence found in kobuviruses from klassevirus, although the increasing sequence identity toward the 5' end of the genome is suggestive that the 5' end may not be complete.

5' RACE products ending at the UUUCGACC sequence shown in Figure 3 were preceded by a poly-dT tract from terminal transferase and two cytosines, which were considered to be from untemplated addition by reverse transcriptase and removed. The first 20 nucleotides have 50% identity with highly conserved kobuvirus sequence that is the 3' end of SL-A and the 5' end of SL-B. Multiple attempts made by RT-PCR to amplify sequence related to the 5' end of a possible SL-A from human klassevirus 1 were unsuccessful.

The klassevirus IRES is considered to be a type II IRES based on the 68% similarity of this region to Aichi virus, however detailed secondary structure analysis did not show a similar IRES structure to that of cardiovirus/aphthovirus [10]. The 5' UTR ends with two in-frame AUG initiation codons at nt 719/722 which are preceded by a 12 nt polypyrimidine tract, with only a 4 nt spacer. The pyrimidine content in this area of the genome was greater than that of Aichi virus or bovine kobuvirus and the spacer region was noticeably shorter than that of other kobuviruses.

### Coding region

The L protein is remarkably short at 111 aa, compared to 170 aa in Aichi virus. Two L protein motifs that have been suggested to be conserved among kobuviruses (PEDxLxDS and LPG) were not present in klassevirus. The 2A protein does not include any H-box/NC protein domains as is apparent in all other kobuviruses as well as some other picornaviruses and does not tblastx with significant similarity to any known sequence [11].

The highest level of sequence identity in the coding region to other picornaviruses (Aichi virus) was found in the 2C, 3D, and VP3 genes. Cleavage sites 2A-2B, 2B-2C, 2C-3A, 3A-3B, 3B-3C, and 3C-3D were all Q-G except 3C-3D which was Q-S. The most conserved region between klassevirus and Aichi virus was in the putative nucleotide binding domain of the 2C helicase (VVYLYGPPGTGKSLLASLLA). A conserved tyrosine was identified in the third position of the 3B-VPg. The conserved 3C protease active site motif that is GXCGG in the enterovirus genus was present but changed to GLCGS, the same as in Aichi virus. The conserved KDELR, YGDD, and FLKR motifs were present in the 3D polymerase [4]. No tandem repeats and no recombination with other picornaviruses were detected in klassevirus.

### PCR screening

Previously described universal kobuvirus primers failed to detect klassevirus in our collection of 751 stool samples (data not shown). New 32-fold degenerate pan-kobuvirus/klassevirus primers were designed to amplify a 200 bp amplicon from the 3D gene and used for RT-PCR screening of 751 stool specimens from symptomatic and asymptomatic individuals from Northern California under code. One additional human klassevirus 1 was detected with screening (2/751, 0.2%). After screening, samples origins were decoded. As it happens, the positive sample was collected from a member of the family that yielded the original sample from which klassevirus was identified. Both children were 11-month old males. Sequence recovered from the 3D gene and 5' UTR was >99% identical between the two samples. No additional kobuviruses or picornaviruses were recovered from the PCR screening using these primers, so it is not known whether these primers are, in fact, pan-kobuvirus/klassevirus primers. The Virochip (v4), a pan-viral microarray designed to detect all known viruses as well as novel viruses on the basis of sequence homology, was unable to detect the novel picornavirus genus. Quantitative PCR from samples 02394-01 and 02394-03 indicated that approximately 5 × 10^7 ^and 1 × 10^7 ^viral genomic copies were present per 1 mL of stool, respectively.

## Discussion

**Figure 4:**
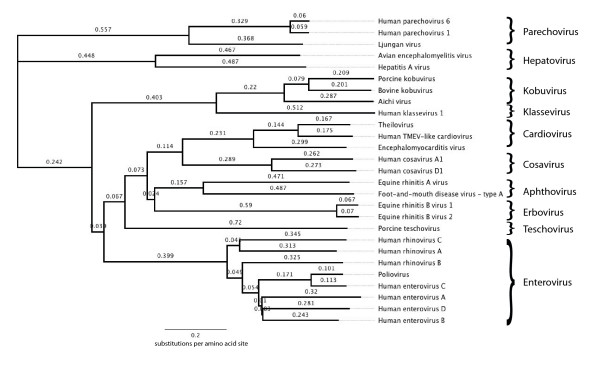
**Phylogenetic tree of klassevirus genome versus strains of other picornavirus genomes from genera based on coding region amino acid identity using clustalw**.

This study presents the discovery and characterization of a novel picornavirus, human klassevirus 1. Klassevirus has a typical picornavirus organization with a ~700–800 bp 5' UTR, long open reading frame and, ~100 bp 3' UTR. The phylogenetic relationship of the new genus to other picornaviruses by amino amino acid sequence is shown in Figure 4. Given that the klassevirus genome possesses <40% amino acid identity in the P1 and P2 regions and <50% amino acid in the P3 region to the nearest picornavirus, this strain qualifies for designation as a new picornavirus genus, as per ICTV standards [12].

Similar to cosavirus, this virus was identified through deep sequencing of stool, a strategy to identify novel viruses that are too divergent to be identified by other methods. Without filtering or selecting for viral particles, we were able to obtain sequence for 75% of the klassevirus genome based on TBLASTX against Aichi virus. Aligning all the pyrosequencing reads to the complete recovered genome of klassevirus indicated that 95% of the viral genome could be identified from the deep sequencing run (Figure 1). This indicates that deep sequencing is a feasible strategy for rapidly identifying entire genomes of novel viruses.

Unlike previously identified kobuviruses, the first 140 nt of human klassevirus 1 is highly divergent. Published studies of Aichi virus suggests the first three stem-loop structures are required for positive and negative strand replication as well as encapsidation [7,8]. The three known kobuviruses share a very high degree of homology in the first 50 bp and all have the three stem-loop structures with pseudoknot originally described in Aichi virus [4]. Multiple attempts were made using 5' RACE to detect the conserved elements at the 5' end of known kobuvirus genomes and all failed. Similar sequence was recovered from both cases of human klassevirus 1 infection and RNAse protection demonstrated that the divergent 5' UTR sequence was part of the klassevirus genome and not an artifact of PCR amplification. We cannot rule out the existence of further 5' nucleotides beyond our current 5' end.

Despite the sequence divergence at the 5' end of its genome compared to known kobuviruses, human klassevirus 1 contains two stem-loops and a pseudoknot structure within the first 140 bp of its genome. Human klassevirus 1 also shares a high degree of sequence identity with Aichi virus throughout the remainder of the 5' UTR, indicating that IRES structure and function is likely preserved between the two viruses. This is especially interesting when compared to porcine kobuvirus which shares the conserved first 50 bp to the kobuvirus 5' UTR but has a hepacivirus/pestivirus-like type IV IRES [4]. Though the exact secondary structure of the Aichi virus and bovine kobuvirus IRES are not known, it has been suggested that they contain type II IRES based on the position of the initial start codon of the polyprotein relative to the upstream polypyrimidine tract [2,10]. The sequence of human klassevirus 1 3' UTR demonstrated almost no homology to other kobuvirus 3' UTR sequences or any other sequence in GenBank.

Although it remains to be determined whether human klassevirus 1 causes bona fide human infection, the data are suggestive. Screening using a newly developed PCR primer pair designed to amplify any klassevirus or kobuvirus found klassevirus only in two young children from the same family. The virus was present in relatively high copy number in both samples, suggesting that replication occurs in the gut and that human klassevirus 1 is not merely a passenger virus. However, both infants were asymptomatic at the time virus was present in their stool. The low prevalence rate is akin to that of Aichi virus, which is a rare known cause human gastroenteritis. Bovine and porcine kobuvirus, on the other hand, have both been found in healthy stool and bovine kobuvirus has been found in the serum of infected cattle [13]. It remains to be determined whether klasseviruses are present in other human tissues or animal hosts.

Future studies to determine a possible link to disease in humans and any unique characteristics of the viral life cycle will be required. Viral culture on human cell lines, especially those from the gastrointestinal tract, could be suggestive that the virus is competent to replicate in human cells and that humans could be a bona fide host of klassevirus. Culture would also help elucidate the importance of different secondary structures in the divergent 5' UTR as well as determine cleavage sites of the polyprotein. Further epidemiological screening and serological assays will be necessary to understand the diversity within this possible genus, the prevalence of klassevirus, and the average age of those infected. Notably, both of the cases in this study were 11 months old, which is approximately the age at which maternal antibodies decline.

## Conclusion

We have detected a new picornavirus genus in stool specimens from two twins and sequenced the viral genome. Further characterization will be required to determine the full extent to which this agent is implicated in human disease, and the spectrum of illnesses to which it may be linked.

## Methods

### Cohort

The cohort has been described previously [14].

### Stool specimen extraction, cDNA amplification, and RT-PCR for genome recovery

Stool suspensions were created by mixing 2 mL of PBS with stool (100–300 mg). 100 uL of stool/PBS mixtures were further diluted in 900 uL of PBS and extracted using the PureLink Viral RNA/DNA 96-well kit (Invitrogen, Carlsbad, CA). Total RNA/DNA was randomly amplified using the round A/B protocol with 25 cycles of PCR before 454 pyrosequencing [15].

Specific RT-PCR was done with Qiagen One-Step RT-PCR kit using 4 uL H2O, 2.5 uL 5× Buffer, 2.5 uL Q solution, 0.5 uL dNTP, 0.5 uL RT/Taq solution, 0.75 uL of F/R 10 uM primer, and 1 uL of stool total RNA. Conditions were 50C for 30 min, 95C for 15 min; 40 cycles of 95C for 30 sec, 50C for 30 sec, 72C for 1 min/kb; and final extension at 72C for 7 min. Degenerate pan-kobuvirus primers targeting the 3D region used for screening were kvF 5'-GYT TTG AYG CYA CCM TYC C-3' and kvR 5'-SGT GTT GAK GAT GGA RGT SSC-3'. Primers for genome recovery are listed in Additional file 1.

3' RACE was done with an adapter-linked oligo-dT primer. Due to problems with secondary structure, 5' RACE was done with a combination of a 5'RACE kit (Invitrogen) and by using the reverse transcriptase activity of Tth polymerase (Promega) at 70C, TdT with 0.2 mM dATP (NEB) for 10 minutes at 37C, and the same adapter-linked oligo-dT primer.

### 454 Pyrosequencing

A total of 141 amplified cDNA libraries that were negative by array and PCR were cleaned via Ampure beads (Agencourt) and quantitated on the Nanodrop spectrophotometer. Aliquots of 200 ng from each sample were combined and sequenced on the Genome Sequencer FLX (Roche) using the Shotgun Sequencing protocol. Sequence analysis of Genome Sequencer FLX data was filtered against human and bacterial sequences using BLAT before unbiased BLASTn and tBLASTx (W3) searches against the BLAST nr database.

### RNAse protection

Total RNA from sample 2394-03 was amplified using 40 cycles of RT-PCR with primers kv1F 5'-CCC TTT CGA CCG CCT TAT-3' and kv761R 5'-CAG CCA ACG AAC TCG AAA AT-3'. The 761 bp amplicon was gel purified and cloned using TOPO TA cloning kit (Invitrogen). TOPO TA plasmid containing the 761 bp insert was sequenced to ensure the correct sequence and insert direction. 1 ug of plasmid was linearized with HindIII and linearly amplified for 10 minutes using MaxiScript (Ambion) kit with 825 nM alpha-P32-UTP and 15 uM unlabeled UTP such that 80 bp of vector sequence flanked both side of the 5' UTR insert. The 920-bp radiolabeled probe was gel-purified on a denaturing 4% polyacrylamide gel following the RPA III kit (Ambion) protocol and quantified using scintillation counting. 80,000 cpm of probe were hybridized with ~50 ng of stool total nucleic acid or aRNA and 5 ug of yeast tRNA and digested with RNase A/T1 using the streamlined protocol from the RPA III kit. The entire sample was run on a denaturing 4% polyacrylamide gel and visualized using a phosphoimager.

### Quantitative PCR

In order to ascertain whether klassevirus underwent replication in the gut or was merely a passenger virus, quantitative PCR was used to determine viral titer in stool. A 134-bp amplicon was generated by RT-PCR using the same conditions above for screening PCR using primers kv3918F/kv4041R and used for standard curve generation (10^9 ^– 10^0 ^copies per reaction). Quantitative PCR was performed on a Mx3005P (Stratagene) under the same RT-PCR conditions listed above for screening PCR, with the exception of Tm 50C for 45 sec, extension at 72C for 45 sec, addition of 1× Sybr Green, and addition of melt curve analysis.

## Competing interests

ALG owns equity in Illumina, Inc.

## Authors' contributions

ALG carried out the initial Virochip and PCR screening, cohort maintenance, 454 sequencing, sequence analysis, full genome recovery, RT-PCR screening, RNAse protection assay, and drafted the manuscript. CR carried out initial Virochip and PCR screening, cohort maintenance, sequence recovery, and sequence analysis. CC carried out maintenance of the cohort and sequence analysis. JP and TD gathered the cohort and organized the data from the cohort. ALG, CR, CC, DG, and JD conceived of the study and participated in its design and helped to draft the manuscript. All authors read and approved the final manuscript.

## Supplementary Material

Additional file 1 **RT-PCR Primers for Klassevirus genome recovery**. Table of RT-PCR primers designed from pyrosequencing reads that were used in this study for klassevirus genome recovery and screening. Click here for file
